# A novel multiplex qPCR assay for clinical diagnosis of non-human malaria parasites-*Plasmodium knowlesi* and *Plasmodium cynomolgi*

**DOI:** 10.3389/fvets.2023.1127273

**Published:** 2023-01-26

**Authors:** Ram Das, Kapil Vashisht, Kailash C. Pandey

**Affiliations:** Parasite-Host Biology Group, ICMR–National Institute of Malaria Research, New Delhi, India

**Keywords:** *Plasmodium knowlesi*, *Plasmodium cynomolgi*, diagnostics, multiplex qPCR, non-human malaria

## Abstract

**Introduction:**

The imminent risk of zoonoses of non-human malaria parasites is not far from reality in India, as has been observed in the case of Plasmodium knowlesi (Pk), and so is possible with *P. cynomolgi* (Pc), already reported from South East Asian countries. Therefore, a novel multiplex qPCR assay was developed and evaluated for detection of non-human malaria parasites- Pk and Pc in populations at risk.

**Methods:**

The qPCR primers were designed in-house with fluorescence labeled probes (HEX for Pk and FAM for Pc). DNA samples of Pk and Pc were used as templates and further the qPCR assay was evaluated in 250 symptomatic and asymptomatic suspected human blood samples from malaria endemic areas of North Eastern states of India.

**Results:**

The qPCR assay successfully amplified the target 18S rRNA gene segment from Pk and Pc and was highly specific for Pk and Pc parasites only, as no cross reactivity was observed with *P. falciparum* (Pf), *P. vivax* (Pv), *P. malariae* (Pm), and *P. ovale* (Po). Standard curves were generated to estimate the limit of detection (LOD) of Pk and Pc parasites DNA (0.00275 & 0.075 ng/μl, respectively). Due to COVID-19 pandemic situation during 2020–21, the sample accessibility was difficult, however, we managed to collect 250 samples. The samples were tested for Pf and Pv using conventional PCR- 14 Pf and 11 Pv infections were observed, but no Pk and Pc infections were detected. For Pk infections, previously reported conventional PCR was also performed, but no Pk infection was detected.

**Discussion:**

The multiplex qPCR assay was observed to be robust, quick, cost-effective and highly sensitive as compared to the currently available conventional PCR methods. Further validation of the multiplex qPCR assay in field setting is desirable, especially from the high-risk populations. We anticipate that the multiplex qPCR assay would prove to be a useful tool in mass screening and surveillance programs for detection of non-human malaria parasites toward the control and elimination of malaria from India by 2030.

## Introduction

In Indian context, major human malaria parasites are *P. falciparum* (Pf), *P. vivax* (Pv), *P. malariae* (Pm)*, P. ovale* (Po) and *P. knowlesi* (Pk) (1–7). Moreover, non-human malaria causing *Plasmodium* species- *P. cynomolgi* (Pc) with established zoonosis in humans has become a cause for concern, at least in the South East Asian countries (8). The risk of expansion of non-human malaria in humans is increasing gradually, majorly due to deforestation, substantial changes in the ecology, host & vector availability and adaptive changes in the parasites (9). It would not be surprising that non-human primate *Plasmodium* parasites- Pk and Pc might be in circulation in the Indian populations, but they are rendered elusive owing to the probable misdiagnosis by routine microscopy and lack of robust diagnostic tools to detect sparsely distributed Pk and Pc infections (10, 11). There are reports of more than 30 species of non-human primate *Plasmodium* spp. and seven of them including Pk, Pc and others- *P. brasilianum, P. eylesi (*Pe*), P. inui* (Pi), *P. schwetzi* (Ps), and *P. simium* had been observed as transmissible to humans (10, 12–18). The major hosts of Pk, Pc, Pi*, P. fieldi* and *P. coatneyi* are the non-human primates *Macaca fascicularis* (long-tailed macaques), *Macaca nemestrina* (the pig-tailed macaque), *Trachypithecus obscuras* (dusky leaf monkey or spectacled langur) and *Presbytis melalophus* (banded leaf monkey) (19, 20). These non-human primate species are prevalent in South East Asia and India as well (21).

The mosquito vectors of human and non-human primate malaria are *Anopheles minimus, An. dirus, An. sundaicus, An. sinensis* and *An. maculates* are commonly found in geographically specific regions of India (1, 5, 22–25). Natural infections of Pk and Pc have been previously reported in macaque monkeys and humans from Malaysia (26, 27). North-Eastern states of India are in proximity to such regions where non-human malaria parasites might be in circulation and humans frequently travel on both sides. India has all the suitable vectors and hosts for non-human *Plasmodium* species; the geographical and climatic conditions are also conducive for the proliferation of Pk and Pc (28).

Pc is phenotypically and phylogenetically similar to Pv; thereby making the identification of Pv and Pc quite difficult in blood slides during routine microscopy. Often, routine microscopy of Pk, Pc and Pm can lead to misdiagnosis by the microscopists in primary health centers of the remote areas. In these circumstances, it becomes imperative to accurately estimate and understand the burden and transmission dynamics of non-human *Plasmodium* spp.- especially Pk and Pc in India human populations.

The current study presents in-house development of a rapid, sensitive and species-specific multiplex qPCR assay targeting the 18S rRNA, for detection of Pk and Pc. Multiplexing for multiple *Plasmodium* parasites (Pk and Pc) in single tube would be efficient to save resources during any kind of mass screening programmes and would also save on precious biological samples. The qPCR assay for Pk was also compared to well-known established PCR assays to detect Pk infections in humans (29).

## Materials and methods

### Design of in-house multiplex qPCR assay

The gene sequences of Pk and Pc 18S rRNA gene were extracted from Reference GenBank accession numbers (Pk-LC483580.1 and Pc-KU708868.1) for design of in-house multiplex qPCR forward and reverse primers and the fluorescence labeled probes (HEX for Pk and FAM for Pc) using online tool (https://biosearchtech.com) (Table 1). The probes were BLAST searched (http://blast.ncbi.nlm.nih.gov/Blast.cgi) and observed to be species-specific for Pk and Pc. The oligonucleotides and probes were commercially synthesized from GCC Biotech (I) Pvt. Ltd., India. The final reaction volume (20 μl) constituted of 4 μl of genomic DNA, 500 nM of each species-specific primers and 400 nM of each probe, 10 μl of 2X Platinum multiplex PCR master mix (Applied Biosystems; Thermo Fisher Scientific, USA). The qPCR conditions were- initial denaturation 95°C for 5 min. followed by 40 cycles of denaturation at-95°C for 15 s. and primer annealing-extension at 53°C for 1 min. The amplifications were performed in BioRad CFX96 Connect Real-Time PCR System, USA.

**Table 1:** List of qPCR primers and respective probes for amplification of Pk and Pc 18S rRNA target regions.

**Primers**	**Sequences (5^′-3^^′^)**
18S PkF	TGCCGACTAGGCTTTGGATG
18S PkR	GGCACTGAAGGAAGCAATCTAAG
Pk Probe	HEX-CTTTTCTCTCCGGAGA-HEX-BHQ1
18S PcF	GCGGTCGCAAATAATGAAGATC
18S PcR	GGGAACAGAAGGAGCGAGAATA
Pc Probe	FAM-TGCTTTTCACGTCAGTGTTTCCAAGA- FAM-BHQ1

### Sensitivity and specificity of qPCR assay

The DNA samples of Pk and Pc parasites were obtained from CSIR-Central Drug Research Institute, Lucknow, India and the yields were observed to be 27 and 7.5 ng/μl, respectively (30). Further, the Pk and Pc genomic DNAs were 10-fold serially diluted four times to estimate the limit of detection (LOD), and each dilution was tested in triplicate. To rule out the cross-reactivity with other species of *Plasmodium* (Pf, Pv, Pm, Po) the assay was validated in 250 blood samples collected from suspected endemic areas of North Eastern States of India. The specificity of Pk was also cross checked using a conventional reported PCR method (29).

### Human blood sample collection and DNA isolation

During 2020-21, the COVID-19 pandemic severely hampered the sample collection. However, a total of 250 samples were collected from north eastern state of India from mass surveys to identify symptomatic and asymptomatic-suspected malaria patients with ethical approval from the Institutional ethics committee (IEC No. ECR/NIMR/EC/ 2019/332). Blood smears were made and blood samples were also collected on Whatman 3MM filter paper for detection of non-human Pk and Pc parasites by microscopy and molecular methods. DNA was isolated from the punched spots using QIAGEN kit as per manufacturer's instructions. All the 250 blood samples were tested for detection of Pf and Pv using nested PCR methods (31).

## Results

**Figure 1:**
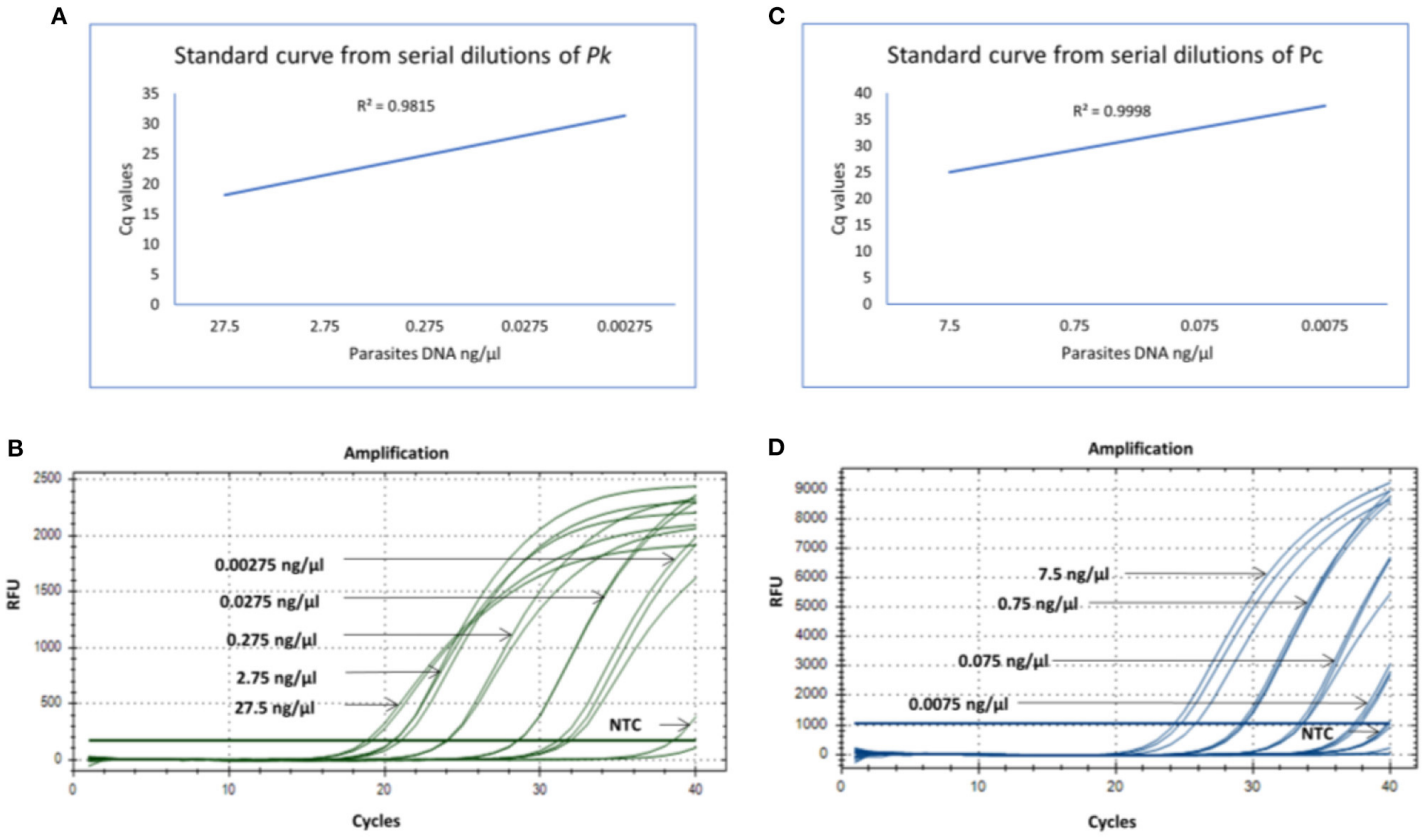
**(A)** Standard curve generated from 10-fold serial dilutions from known concentrations of parasites DNA (Pk) in the qPCR assay. **(B)** qPCR amplification cycles for Pk serial dilutions with fluoroscent probe HEX (green). **(C)** Standard curve generated from 10-fold serial dilutions from known concentrations of parasites DNA (Pc) in the qPCR assay. **(D)** qPCR amplification cycles for Pc serial dilutions with fluoroscent probe FAM (blue).

**Figure 2:**
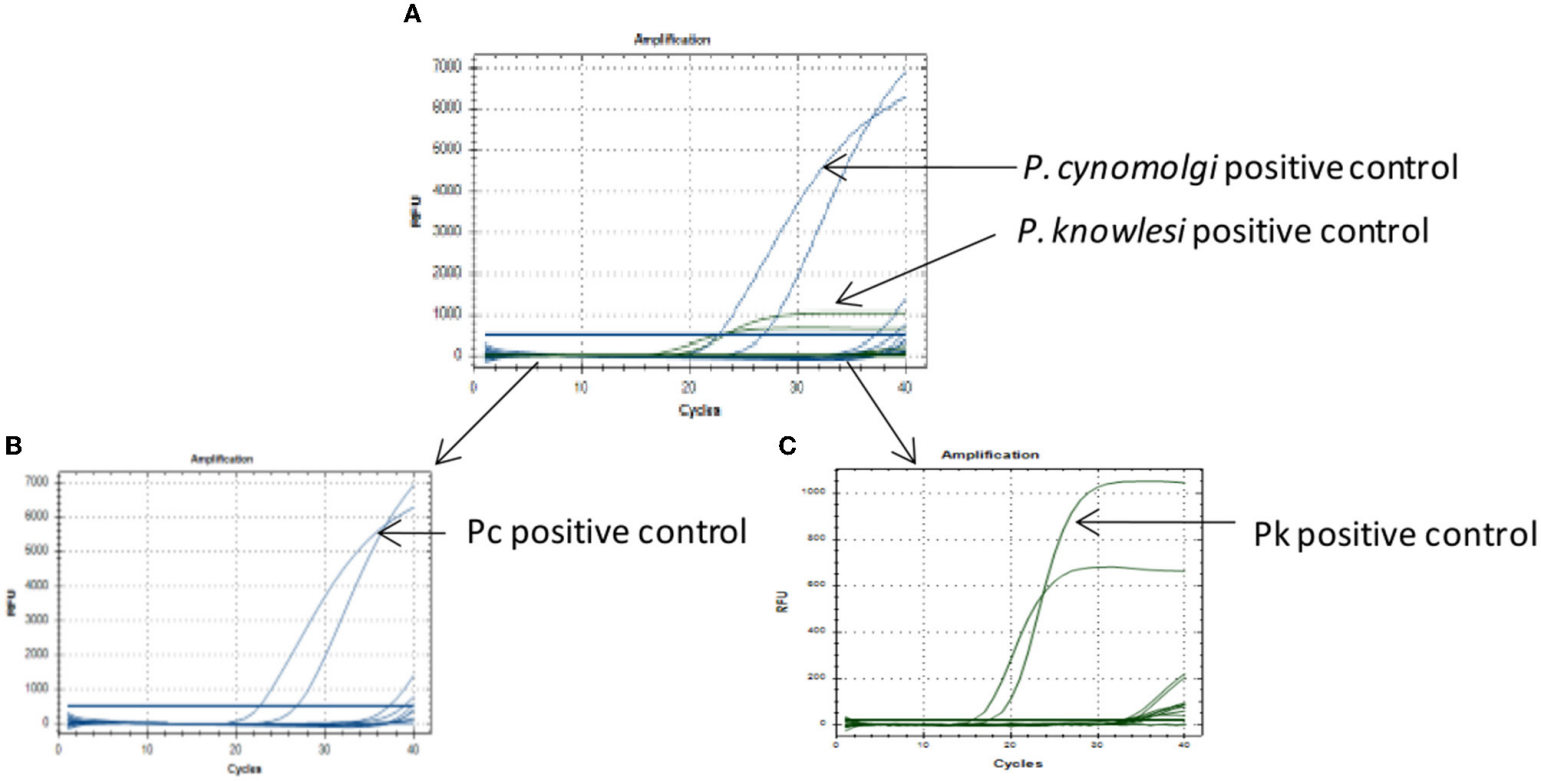
Specificity of the qPCR assay. **(A)** Multiplex qPCR assay for detection of Pk and Pc parasites DNA as positive controls, while templates from Pf, Pv, Pm and Po parasites DNA did not amplify. **(B)** Selected fluorophore probe FAM (blue) for *P.cynomolgi* showed good amplification but no amplification for Pf, Pv, Pm, and Po. **(C)** Selected fluorophore probe HEX (green) for *P. knowlesi* showed good amplification but no amplification for Pf, Pv, Pm, and Po.

The qPCR assay was designed for detection of 18S rRNA gene segment of the Pk and Pc parasites. The in-house designed primers and probes were used for the amplification of the 18S rRNA gene segments of the Pk and Pc, respectively. Standard curves from the serial dilutions were generated for each parasite (Pk and Pc) with estimated R^2^ values of 0.98 and 0.99, respectively (Figures 1A, C). To assess the limit of detection (LOD) of parasites DNA, the Pk and Pc DNA were serially diluted and subjected to qPCR assay. The LOD of parasites DNA in multiplex qPCR assays were observed to be 0.00275 and 0.075 ng/μl of Pk and Pc genomic DNA, respectively (Figures 1B, D). The associated C_q_ values corresponding to the minimal detection limits were 28.50 and 31.5 for Pk and Pc, respectively (Figures 1B, D). The in-house designed qPCR primers and probes were observed to species-specific for Pk and Pc in the multiplex format, as the 18S rRNA primers and probes specific for Pk and Pc did not amplify any of the human malaria parasites DNA (Pf, Pv, Pm and Po) (Figure 2); thus, validating the multiplex qPCR assay to be species-specific for Pk and Pc.

Microscopy was used for detection of malaria parasites and further, the multiplex qPCR primers and probes were evaluated with 250 human blood samples collected from suspected areas from North Eastern states of India. We did not observe any Pk and Pc positive infection from these samples. However, we did observe Pf and Pv infections using microscopy and previously reported primers for detection of Pf and Pv parasites (31) in these samples as listed in Table 2. 27/250 samples were febrile (symptomatic) at the time of sample collection; males and females were in approximately equal proportion of the total number of samples. 14/250 samples were found to be positive for Pf infections; while 11/250 were found positive for Pv infections. However, none of the samples were observed positive for Pk and Pc parasites, either by the in-house developed qPCR assay as well as with established conventional PCR primers described previously for detection of Pk (29). In the absence of any positive sample for Pk at the least even by the conventional PCR primers reported previously, the qPCR primers for Pk would be considered highly sensitive, however further validation of the primers shall be assessed with Pk positive samples from other sites in future.

**Table 2:** Age-group wise infections of Pf and Pv in symptomatic/asymptomatic patients.

**Age groups (years)**	**No. of samples**	**Males**	**Females**	**Febrile (Symptomatic)**	**Febrile cases**		**Afebrile cases**	
					**Pf**	**Pv**	**Pf**	**Pv**
01–09	82	41	41	17	4	5	0	0
10–19	51	29	22	3	1	2	0	2
20–29	48	23	25	0	0	0	5	0
30–39	24	13	11	2	0	0	0	1
40–49	23	11	12	0	0	0	3	1
50–59	11	4	7	5	1	0	0	0
>60	11	6	5	0	0	0	0	0
Total	250	127	123	27	6	7	8	4

## Discussion

The non-human *Plasmodium* parasites Pk, Pc, *P. fieldi* and *Pi* have been reported from Malaysia; while Pk and Pc were also reported from India as well (4–8). The non-human malaria parasite Pk in the human host and vector have been reported from different states of India such as Bihar, Delhi, Andaman & Nicobar Islands and Uttar Pradesh (1, 2, 5, 7). In this study, we developed in-house multiplex qPCR assay for detection of non-human malaria parasite species Pk and Pc. This diagnostic tool shall prove to be critical in detection, identification, surveillance and monitoring of the non-human malaria parasite Pk and Pc, ultimately contributing toward the control and elimination of malaria by 2030 from India.

The lowest limit of detection from genomic DNA of Pk and Pc parasites DNA was found to be 0.00275 and 0.075 ng/μl, respectively (Figures 1B, D). The probes were also BLAST searched and found to species-specific for Pk and Pc only. Further, no cross-reactivity was observed with any of the human malaria parasites- Pf, Pv, Po and Pm, proving them to be species-specific (Figure 2). Therefore, the in-house developed species-specific multiplex qPCR assay is highly specific and sensitive for detection of Pk and Pc. The assay was further evaluated for detection of Pk and Pc in 250 human blood samples collected from highly malaria endemic areas of North Eastern states of India and yielded no positive infection for Pk and Pc.

We anticipate that the qPCR assay would prove to be a useful tool for detection of Pk and Pc infections in the vector mosquitoes also (*An. dirus, An. minimus, An. sundaicus, An. sinensis* and *An. maculates*) as well as in their natural hosts- non-human primates (1, 5, 21, 23–25, 28). The multiplex qPCR assay for detection of Pk and Pc parasites is robust, quick, cost-effective, sensitive and species-specific to undertake investigations particularly focused on the transmission dynamics of these non-human malaria parasites in India. However, further studies with larger number of samples are needed to validate the usefulness of the qPCR assay for Pk and Pc.

## Data availability statement

The raw data supporting the conclusions of this article will be made available by the authors, without undue reservation.

## Ethics statement

The studies involving human participants were reviewed and approved by Institutional Ethics Committee ICMR-National Institute of Malaria Research, Delhi, India (IEC No. ECR/NIMR/EC/ 2019/332). Written informed consent to participate in this study was provided by the participants' legal guardian/next of kin.

## Author contributions

RD conceived the study, designed and performed the experiments, analyzed the data, and wrote the manuscript. KV analyzed the data and wrote the manuscript. KP analyzed the data and reviewed the manuscript. All authors contributed to the article and approved the submitted version.
